# Historical Perspective and Biotechnological Trends to Block Arboviruses Transmission by Controlling *Aedes aegypti* Mosquitos Using Different Approaches

**DOI:** 10.3389/fmed.2020.00275

**Published:** 2020-06-23

**Authors:** Marina Luiza Rodrigues-Alves, Otoni Alves de Oliveira Melo-Júnior, Patrícia Silveira, Reysla Maria da Silveira Mariano, Jaqueline Costa Leite, Thaiza Aline Pereira Santos, Ingrid Santos Soares, Daniel Ferreira Lair, Marília Martins Melo, Lucilene Aparecida Resende, Denise da Silveira-Lemos, Walderez Ornelas Dutra, Nelder de Figueiredo Gontijo, Ricardo Nascimento Araujo, Mauricio Roberto Viana Sant'Anna, Luis Adan Flores Andrade, Flávio Guimarães da Fonseca, Luciano Andrade Moreira, Rodolfo Cordeiro Giunchetti

**Affiliations:** ^1^Laboratório de Biologia das Interações Celulares, Departamento de Morfologia, Instituto de Ciências Biológicas, Universidade Federal de Minas Gerais, Belo Horizonte, Brazil; ^2^Departamento de Clínica e Cirurgia Veterinárias, Escola de Veterinária, Universidade Federal de Minas Gerais, Belo Horizonte, Brazil; ^3^Departamento de Medicina, Universidade José Do Rosário Vellano, UNIFENAS, Belo Horizonte, Brazil; ^4^Laboratório de Fisiologia de Insetos Hematófagos, Departamento de Parasitologia, Instituto de Ciências Biológicas, Universidade Federal de Minas Gerais, Belo Horizonte, Brazil; ^5^Laboratório de Virologia Básica e Aplicada, Departamento de Microbiologia, Instituto de Ciências Biológicas, Universidade Federal de Minas Gerais, Belo Horizonte, Brazil; ^6^Laboratório de Mosquitos Vetores: Endossimbiontes e Interação Patógeno-Vetor, Instituto René Rachou, Fiocruz, Belo Horizonte, Brazil

**Keywords:** *Aedes aegypti*, arboviruses, vector control, Latin America, vaccines

## Abstract

Continuous climate changes associated with the disorderly occupation of urban areas have exposed Latin American populations to the emergence and reemergence of arboviruses transmitted by *Aedes aegypti*. The magnitude of the financial and political problems these epidemics may bring to the future of developing countries is still ignored. Due to the lack of effective antiviral drugs and vaccines against arboviruses, the primary measure for preventing or reducing the transmission of diseases depends entirely on the control of vectors or the interruption of human-vector contact. In Brazil the first attempt to control *A. aegypti* took place in 1902 by eliminating artificial sites of eproduction. Other strategies, such as the use of oviposition traps and chemical control with dichlorodiphenyltrichlorethane and pyrethroids, were successful, but only for a limited time. More recently, biotechnical approaches, such as the release of transgenics or sterile mosquitoes and the, development of transmission blocking vaccines, are being applied to try to control the *A. aegypti* population and/or arbovirus transmission. Endemic countries spend about twice as much to treat patients as they do on the prevention of mosquito-transmitted diseases. The result of this strategy is an explosive outbreak of arboviruses cases. This review summarizes the social impacts caused by *A. aegypti*-transmitted diseases, mainly from a biotechnological perspective in vector control aimed at protecting Latin American populations against arboviruses.

## Introduction

Mosquitoes of the Culicidae family are considered the most dangerous animals on earth due to their capacity to transmit diseases and their medical importance regarding viruses, protozoa, and nematode transmission (1). According to the Pan American Health Organization (PAHO) vector-borne diseases are considered public health problems and have a major social and economic impact causing high morbidity and mortality, especially in developing countries (2). Approximately 75% of the Latin American population lives in cities, which makes the most urbanized continent in the world (3). The magnitude of climate and ecosystem changes and disorderly occupation of urban areas may increase the vulnerability of human populations to infectious disease transmission (2). Moreover, these changes have led several vectors to adapt to the urban environment (4, 5). We list the top 15 pathogens transmitted by Culicidae in the Americas and the prevalence of viruses is evident (80%), all considered as arboviruses (Table 1). *Aedes* genus has drawn the attention of the world health authorities due to the severity of diseases transmitted in the last 10 years, especially Dengue, Chikungunya, and Zika fevers.

**Table 1:** Culicidae family includes vectors of major medical importance and the main pathogens transmitted in the Americas.

**Vector**	**Pathogen**	**References**
*Aedes*	Dengue vírus	(6)
	Yellow fever vírus^*^	(7)
	Chikungunya vírus	(8)
	Zika vírus	(9)
	La Crosse vírus	(10)
*Anopheles*	*Plasmodium falciparum*	(11)
	*Plasmodium vivax*	(12)
	Guaroa vírus	(13)
*Culex*	*Wuchereria bancrofti*	(14)
	West Nile vírus	(15)
	Saint Louis Encephalites virus	(16)
	Estern Equine Encephalites virus	(17)
	Wester Equine Encephalites virus	(17)
*Culicoides*	Oropouche virus	(18)
*Haemagogus*	Mayaro virus	(19)

* *The Yellow fever virus transmission in urban areas in Brazil has been interrupted since 1942*.

The *Aedes* genus has more than 950 species of mosquitoes, many of which transmit potentially deadly pathogens to humans and other animals. Among the species, *Aedes aegypti* is currently the main global arbovirus vector (20). Carvalho and Moreira (21) reviewed the main reasons for *A. aegypti* having both reproductive success and being so well-adapted to the urban environment. This mosquito lives in urban habitats and reproduces primarily in artificial containers (5, 6, 21, 22). A single female makes several blood meals during each feeding period and hematophagy occurs preferentially in humans (21). The blood meal is essential for egg maturation. During oviposition, the female spreads the eggs in many breeding sites to ensure reproductive success and can remain viable for over 1 year (20, 21). Beyond the vertical transmission, an infected female is able to transmit the arbovirus for the rest of its life after virus incubation (21). In this review, we demonstrate the impact caused by *Aedes aegypti*-borne diseases based on current literature, highlighting the history of vector control based on the major biotechnological approaches for preventing new arboviral epidemics.

## Origin of the Main Arboviruses That Cause Human Diseases and the Role of *Aedes aegypti* in Their Transmission

Arbovirus (arthropod-borne virus) is the non-taxonomic term used to define a group of viruses transmitted by hematophagous arthropods (23). According to the International Catalog of Arbovirus, there are 545 species and most of the viruses registered have non-human vertebrates as the main reservoir (6, 22, 24). It is believed that approximately 150 arboviruses cause diseases in humans, most of them zoonotic, and belonging to five viral families, namely Bunyaviridae, Flaviviridae, Reoviridae, Rhabdoviridae, and Togaviridae (6, 24). In humans, clinical manifestations of arboviruses include moderate or severe febrile illness with or without hemorrhage, headache, retro-orbital pain, rash, myalgia, arthralgia, and several neurological syndromes (24). Currently, international travel and the global distribution of vectors have made arboviruses a constant threat to the human population (2, 24). The reasons for the emergence or reemergence of some diseases are not fully known, but the following mechanisms have been identified as causes of change or increases in the incidence of many diseases: altered habitat, loss of biodiversity, niche invasion or host–shifting by pathogens, human–induced genetic changes in disease vectors or pathogens, environmental contamination, and diminishing global travel times, when infected people carry pathogens before symptoms occur (2, 25, 26).

**Figure 1:**
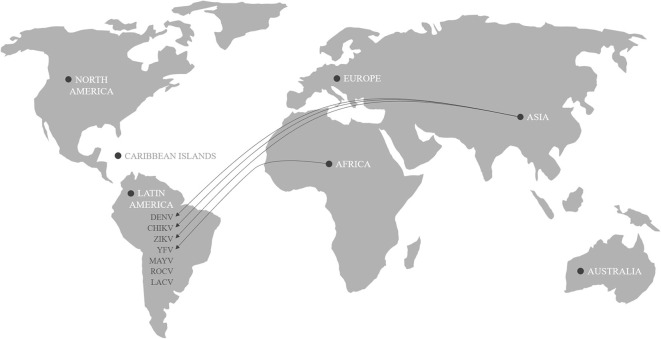
Origin of arboviral genotype of Dengue (DENV), Chikungunya (CHIKV), Zika (ZIKV), Yellow Fever (YFV), Mayaro (MAYV), Rocio (ROCV), and La Crosse virus (LACV) causing human diseases transmitted by *Aedes aegypti*.

*A. aegypti* was the main vector of some of the most important arboviral epidemics of all time such as Yellow fever virus (YFV) and dengue virus (DENV), which resulted in millions of fatalities worldwide (6, 22, 27). In addition to the extensive history of epidemics, chikungunya virus (CHIKV) and Zika virus (ZIKV) caused serious outbreaks in the Americas continent in recent years (9, 28–30). The major arboviruses causing human diseases transmitted by *Aedes* mosquitoes in the Americas and their possible places of origin are described in Figure 1.

The YFV came from West Africa and arrived in the Americas during the slave trade in the sixteenth and seventeenth centuries (30). It is believed that the DENV spread throughout the world through transport routes in the eighteenth and nineteenth centuries and that, after establishing itself, decreased between the 1950s and 1970s due to the aggressive vector control method of the time (6, 29, 31). In the 1990s, DENV reemerged following a decline in vector control efforts and increased insecticide resistance, culminating in one of the worst global epidemics with 2,326,000 reported cases in the Americas in 2015 (24, 28). The first case of CHIKV on the Caribbean island of Saint Martin was confirmed in October 2013. Subsequently, CHIKV spread to the Caribbean, Central America, and some regions of South America, totaling about 1.6 million cases (28). Chikungunya fever is a debilitating disease characterized by high fever associated with chronic polyarthralgia and generalized rash (23, 28, 32). Some complications have been reported, including hemorrhaging and cardiac, neurological, and gastrointestinal injury (22, 32). The social damage caused by CHIKV infection is inestimable because the virus can remain in the tissues of the joints, causing persistent arthralgia for months or even years (23). Kantor (23) suggested the virus was imported from Asia since it was shown that the isolates had the Asian genotype. Oliveira Melo et al. (33) suggested that ZIKV introduced in the Americas also came from Asia. This study evaluated two pregnant women from the state of Pernambuco, in northeast region of Brazil, who were diagnosed with fetal microcephaly and had symptoms of ZIKV infection. The patients were submitted to an amniocentesis test, blood test, and qPCR to detect the presence of the virus. Although the blood test was negative, amniocenteses and qPCR showed a positive result for ZIKV. The virus was isolated and sequenced, and the result showed Asian genotype in both cases (33). Studies based on the sequence of a large number of virus isolates indicated that a cryptic ZIKV circulation occurred in the Northeast of Brazil as early as February 2014, and from the region the virus disseminated nationally and internationally (34). In February 2016, the World Health Organization declared the situation “a public health emergency of international importance” due to the increasing number of cases of ZIKV infection in Brazil, and its possible association with neurological disorders and congenital anomalies, such as microcephaly (22, 29, 33). In 2016, the situation worsened and at the end of the year, the World Health Organization estimated that ~4 million people were infected with ZIKV. The impact of this number of cases in Brazil had deeper social and emotional consequences, since 1,434 cases of microcephaly were confirmed (2). The extent of the financial and political problems that the epidemic may cause in the future is still unknown, but efforts need to be concentrated on supporting and monitoring affected families. Lately, the first clinical case of infection with Mayaro (MAYV), a virus of the Togaviridae family recently arrived in Haiti, has been described. This virus was discovered in 1954 in Trinidad and Tobago, but so far only isolated cases are known in the Amazon and other regions of South America. According to epidemiological data available to the Ministry of Health, there were 197 notifications distributed in nine Brazilian states between December 2014 and June 2015, mainly in the north and center-west regions. There are still no records of deaths from the disease, but as is the case with CHIKV, those infected can continue to experience joint pain for weeks or months. Although *Haemagogus* sp. is the main vector of MAYV, *A. aegypti* is also capable of transmitting this arbovirus (35). The constant transit of arboviruses represents a challenge in the diagnosis and treatment of patients because, in addition to causing similar clinical manifestations (36), they can be transmitted simultaneously.

Along with the five arboviruses listed above, it is worth highlighting the presence of La Crosse and Rocio viruses, which can be transmitted by mosquitoes of the genus *Aedes*. The first cases of Rocio virus (ROCV) were recorded in the southeastern region of Brazil during a viral encephalitis epidemic that lasted for about 2 years in the 1970s. Although subsequent outbreaks did not occur, there was serological evidence of ROCV circulation, which serves as a warning signal for possible new epidemics (37). La crosse virus (LACV) is present in Latin America and in some regions of North America and is transmitted by mosquitoes of the genus *Aedes*. Although *A. aegypti* is not a very efficient vector, *A. albopictus* is able to transmit this virus. The cases of encephalitis due to LACV infection have low mortality, but there are reports of patients with neurological complications, which may make the differential diagnosis difficult with other arboviruses (10).

## Importance of *Aedes aegypti* and Biotechnological Approaches Applied for Mosquito Control as a Strategy to Prevent the Emergence of Arboviruses

### Physical and Chemical Control Approaches

The physical and chemical control methods are based on the elimination of breeding sites and in the use of insecticides to reduce population density, thus decreasing the transmission of pathogens and, consequently, preventing epidemics. In 1902, Oswaldo Cruz led the first Brazilian public campaign to control *A. aegypti*, which was based on the elimination of artificial breeding sites. At the time, this campaign aimed to reduce the transmission of yellow fever. However, the vector was eradicated only in the middle of 1955. Afterwards, the mosquito was reintroduced over time and became endemic in much of the country. In the following years, control strategies were guided by the “Programa Nacional de Controle da Dengue” (PNCD), which recommended using physical strategies based on the identification of regions according to infestation rates. To date, WHO has promoted integrated vector control, in addition to conventional tools such as mosquito nets treated with materials, nets, oviposition traps (ovitraps), among others. Numerous studies have shown that ovitraps are an effective strategy that contributes to the monitoring of *A. aegypti* populations hence the areas at risk of transmission of arboviruses (38–43). Mackay et al. (44) developed the autocidal gravid ovitrap (AGO-A), a lethal ovitrap based on the gravid ovitrap (GO). It has already been shown that the use of AGO has significantly reduced the prevalence of mosquitoes infected with CHIKV (39). The main limitation to using an ovitrap is that it contains insecticides, which may not be effective in regions where the mosquito population is resistant.

The larval period is the only immature stage during which feeding and growth occur in the life cycle of *A. aegypti*. The chemical control measures employed preferentially focus on this stage due to three striking attributes. First, the larvae are aquatic and feed on suspended organic matter adhering to the walls or sediment at the bottom of the reservoirs. As larval eating habits are not selective, they can easily ingest chemical or biological insecticides (45). Second, the larvae breathe atmospheric air through the siphon, located in the eighth abdominal segment. The moment they rise to the surface to breathe, they become more susceptible to control agents (21). Third, they are restricted to the oviposition site, which restricts their ability to migrate (21, 45). The main insecticides used act on the insects'central nervous system and belong to the groups of organochlorines, carbamates, organophosphates, and pyrethroids (46, 47). The indiscriminate use of Dichloro-diphenyltrichlorethane (DDT) and pyrethroids induced the development of resistance, which negatively impacted the effectiveness of vector control interventions in endemic countries (48–52). Macoris et al. (53) described that, after 10 years without usage of pyrethroids in São Paulo state, Brazil, *A. aegypti* showed persistent resistance. A bioassay with papers impregnated with a deltamethrin diagnostic dose (DD) was conducted yearly, from 2004 to 2015, with *A. aegypti* adults from 7 different sites, showing resistance in most cases. These results were a direct effect of pyrethroids being extensively used by São Paulo state governments between 1989 and 2000, and being the preferred insecticide for domestic use (53). This scenario is not limited only to Brazil or South America. Moyes et al. (54) described that resistance to pyrethroids are widespread throughout the world. Temephos was the preferred larvicide used in Brazil from 2003 to 2014. As a result of continuous usage, cases of resistance were detected in most of studies in all Brazilian geographic regions as compiled by Valle et al. (55). Corte et al. (56) conducted a study from October 2010 to August 2011 to evaluate *A. aegypti* resistance to Temephos from 7 municipalities in Sergipe state, Brazil. All mosquitoes were found to be resistant, varying between cities, even with an increased dose of Temephos. In recent years, interest has grown in the identification of plants with insecticidal properties (57). The use of plant extracts in the control of *A. aegypti* demonstrated low production cost, high biodegradability, and different active elements that delay the development of resistance in insects (57–62). However, phytochemicals can also have toxic effects that vary according to the species of the plant, with the part used, age of the plant, and extraction with selected solvent (57, 58, 63).

### Immunoprophylactic and Biological Control Approaches

Several research groups around the world have focused on the search for arbovirus vaccines, primarily against DENV (64–69). Generally, this type of approach is species-specific and considering the huge variety of known arboviruses, vaccine strategies that seek to prevent transmission seem difficult and remote. This is a long process that requires active participation of public policies and a significant financial investment, which does not occur in most endemic regions (70–72). Several DENV vaccine candidates have been developed over the years, some of which are in the testing phase. The composition of the formulations varies between DNA (monovalent or tetravalent), recombinant adenoviruses, Alfavirus replicons, and chimeric E protein subunits, among others (64–69, 73, 74). However, only the CYD-TDV vaccine has reached the final stages of testing in humans in different parts of the world (75). CYD-TDV (Sanofi-Pasteur) is an attenuated chimeric vaccine containing recombinant fractions from the four serotypes of DENV (tetravalent). The tetravalent attenuated component (TDV) was developed by the Reed Army Research Institute (WRAIR), in collaboration with GlaxoSmithKline Vaccines (68). Sanofi Pasteur then developed the vaccine including the viral strains PUO-359 (DENV-1), PUO-218 (DENV-2), PaH881/88 (DENV-3), and 1228 (DENV-4) (75). After going through the clinical study phases, the vaccine was made commercially available under the name DengVaxia® (CYD-TDV, Sanofi-Pasteur). According to da Costa et al. (76), the effectiveness of this vaccine varies around 59%, but more studies are needed to confirm its long-term effectiveness. However, on December 13, 2017, WHO issued a statement warning that DengVaxia® should not be administered to people who have not been previously exposed to DENV due to an increased incidence of childhood cases of severe dengue in vaccinated individuals who had never been infected with the virus. Still, it remains the only arbovirus vaccine available at the moment.

While efforts in the search for vaccine targets are being made, vector control strategies need to be developed that can prevent the emergence of new epidemics and the simultaneously control circulating arboviruses. Due to the lack of antiviral drugs against arboviruses, the main measure for preventing or reducing the transmission of diseases depends entirely on the control of vectors or the interruption of human-vector contact (27). In this context, the use of sterile insect technique (SIT) and Release of Insects carrying a Dominant Lethal (RIDL) developed by genetic engineering was proposed as a control strategy (77). Alphey and Andreasen (78) reviewed these techniques, which are largely based on the RIDL. After copulation, this dominant gene causes the death of its progeny. These approaches show a great potential for disease control (79, 80) and have been tested in different endemic countries for *A. aegypti* control (81–84). In Brazil, the use of OX513A self-limiting strain reduced the local *A. aegypti* population by up to 95% in a suburb of Juazeiro, Bahia (85). This transgenically modified *A. aegypti* mosquito containing a dominant lethal gene transgenic strain (OX513A), was incorporated into the target field population (86). Furthermore, the impacts of introgression from a *A. aegypti* transgenic strain remain unclear for arbovirus control and transmission purposes (86).

A number of natural predators (fish and crustaceans) and pathogenic organisms (fungi and bacteria) have already been employed in an attempt to control *A. aegypti*. The entomopathogenic bacteria Bacillus thuringiensis var. Israelensis (Bti) is commonly found in nature and carries toxins with strong insecticidal activity through the formation of spores and crystals. The Bti has high specificity for *A. aegypti* and is practically innocuous to humans (21, 87). In addition, the combined action of different toxins reduces the risk of resistance (88). The use of this bacteria enabled the development of larvicides such as DengTech® (Fiocruz/BR3) and VectoBac WG. However, it has been reported that environmental factors such as high temperatures and a high annual rainfall interfere with VectoBac's efficacy (89).

An interesting approach is the use of *Wolbachia* gram-negative bacteria (Order Rickettisiales) discovered by Hertig and Wolbach (90) in the reproductive tissues of *Culex pipiens*. It is known that the different strains establish symbiotic relationships with more than 60% of the world's insect species and that they have a great potential to reduce the life span of mosquitoes and block infection by pathogens (27, 91–96). This strategy can be used as a population suppression or as a population replacement approach. The latter uses strains of *Wolbachia* that can block arboviruses, especially Flaviviruses (27, 97) and Alphaviruses (98). Several studies on this approach have shown promising results, especially in Australia (27, 92, 94–100). McMeniman et al. (101) showed that the introduction of the wMelPop-CLA, a *Wolbachia* strain, reduced a population of *A. aegypti* kept in the laboratory by up to 50%. Moreira et al. (27) demonstrated that the introduction of this strain inhibits the infection of DENV-2 and CHIKV in *A. aegypti* and suggested that the blocking strategy, in synergy with the reduction in life span, could be a promising approach to controlling the transmission of arbovirus. Walker et al. (96) transfected mosquitoes with the avirulent wMel, another *Wolbachia* strain, and showed a more accelerated invasion of *A. aegypti* populations when compared to the wMelPop-CLA strain in semi-field conditions. The authors also showed a blockage of DENV-2 transmission by both strains. The use of *Wolbachia* as a vector control method provided subsidies for the creation of the “Eliminate Dengue: Our challenge” Project (World Mosquito Program—WMP). The release of mosquitoes in northern Australia (2011) showed that *Wolbachia* had already spread throughout the population of *A. aegypti* 10 weeks later. The WMP originally aimed to control the transmission of DENV, but today it contributes significantly to the control of other arboviruses in 12 countries, including Brazil. Currently, it is being used in Brazil by the World Mosquito Program (formerly “Eliminate Dengue: Brazil Challenge”) and has shown results worth of attention (102). Moreover, the use of this bacteria has also proved to be a promising method of controlling other viruses. Ekwudu et al. (103) evaluated the ability of the wAlbB strain to reduce or block the replication of Flavivirus and Alphavirus species in cell cultures and showed that this control strategy is effective against a wide variety of RNA viruses. The cultures in the presence of WNV, ZIKV, Ross River (RRV), Barmah Forest (BFV), and Sindbis virus (SINV) infected with wAlbB showed significantly reduced in viruse load compared to *Wolbachia*-free controls.

Despite abundant evidence of success, the use of *Wolbachia* presents limitations in countries with wide temperature fluctuations. Ross et al. (104) showed a drastic reduction in the frequency of wMel infection after a heatwave of a 43.6°C, an effect that is stage specific. Another limitation of this strategy is that the establishment of *Wolbachia* in the mosquito population depends on resistance to insecticides. Garcia et al. (105) released *A. aegypti* strains infected with *Wolbachia* using susceptible (wMelBr) and resistant (wMelRio) mosquito-infected *Wolbachia* strains to pyrethroids in an isolated region of Rio de Janeiro, Brazil. Only the *A. aegypti* strain infected with the resistant strain (wMelRio) allowed the establishment of *Wolbachia*. The use of *Wolbachia*, when combined with other strategies such as a transmission blocking vaccine (TBVs), could help reduce arbovirus transmission; yet endemic countries spend about twice as much on treating patients as they do on preventing mosquito-transmitted diseases (106). The cost of dengue in the Americas is already billions of dollars a year (106). The strategy of investing in patient treatment and disregard vector control has already proven to be a complete failure, as arbovirus-related diseases have shown no clear sign of reduction over the past years.

**Figure 2:**
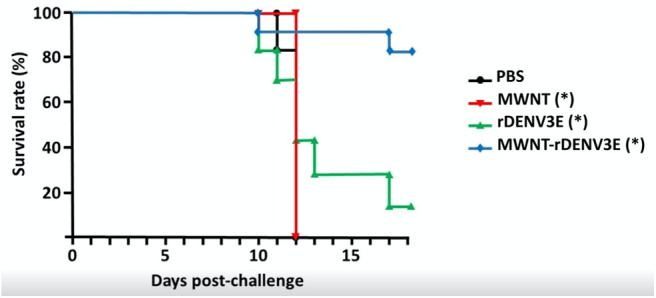
Survival rates in mice immunized or not with a subunit anti-Dengue vaccine and challenged with a virulent DENV3 strain. Briefly, the recombinant Dengue serotype 3 Envelope protein was produced in *E. coli*, purified by affinity chromatography, and quantified. Purified recombinant proteins were covalently linked to multi-walled carbon nanotubes though a diimide-activated amidation protocol (115). Evaluation of the nanotubes functionalization was verified by transmission electronic microscopy and Raman spectroscopy. Ten-weeks old BALB/c mice were divided into 4 groups: non-immunized control (PBS), vaccine-immunized group (MWNT-rDENV3E), animals immunized with the recombinant protein alone (rDENV3E), and animals inoculated only with pristine nanotubes (MWNT). All groups were either immunized or mock-immunized three times every 7 days, through the intramuscular route. After 7 days of the last inoculation, animals were challenged with 10^3^ PFUs of DENV3. Animals were evaluated daily until the appearance of neuropathology and were then humanely euthanized when a 25% weight loss was confirmed. In total, surviving mice were evaluated for 21 days. The study was approved by the Committee on the Ethics of Animal Experiments from the Universidade Federal de Minas Gerais (CETEA/UFMG 270/2010). The symbol “*” in the groups (MWNT, rDENV3E, MWNT-rDENV3E) demonstrates statistical differences (*p* < 0.05) compared to PBS group, according One-way ANOVA test and Tukey's post-test analysis.

The TBVs are strategies that consist of using essential vector proteins capable of inducing specific antibody production after host immunization. Therefore, after blood meal on an immunized host, the antibody triggered by vaccination could induce interference in the cell cycle on the vector or/and block virus infection to the vector, thus preventing disease transmission to human hosts (107). Some successful examples against transmission of *Plasmodium berghei* by *Anopheles stephensi* (108), *P. vivax* (109) by *A. dirus* (110), *P. falsiparum* by *A. gambiae* (111), and *Leishmania infantum* by *Lutzomyia longipalpis* (112) have been reported in the literature, showing this strategy as promising and possibly helpful in interrupting distinct pathogen transmission cycle. Notwithstanding the potential of TBVs, they do not directly protect humans, so there is resistance to their use in public health programs (113). In 1980, there were about 95 species of arboviruses cataloged in Brazil alone, but this number has doubled in the last three decades and today 210 arboviruses circulate in the country. It is known that at least 37 of them are capable of causing disease in humans (2). The recent epidemics in South America warn of the possibility of the emergence of new arboviruses and the resurgence of diseases transmitted by previously controlled vectors (114). In addition to preventing disease transmission, new strategies for sustainable control of vector populations are highly recommended (27). As viruses first infect and multiply in the midgut, molecules overexpressed after blood meal have been suggested as possible TBVs. The challenge with this strategy is the fact that some viruses have more than one serotype (114). Our research group has focused on the development of TBVs against some vectors, including *A. aegypti*. In a pre-clinical trial, we analyzed distinct vector antigen targets. The data are in the final phase of analysis, but we have already identified promising targets that interfered in all *A. aegypti* developmental stages and reduced the number of mosquitoes generated at the end of the first generation by up to 90% (unpublished data). TBVs that target specific mosquito molecules can help to reduce the circulation of pathogens transmitted by the same vector simultaneously. Taking into account the challenges of controlling vector-borne diseases, the future of vaccines against pathogens transmitted by vector will most likely incorporate the TBV rationale. One example is the use of subunit vaccines against Dengue in which nanocarrier platforms are added to recombinant immunogenic DENV proteins. Such nanocarriers are able to increase the immunogenicity of rather poor immunogens in the presence of adjuvants, potentiating their use. Carbon nanotubes are a good example of carriers that could be used for this purpose (Figure 2), being also associated to the TBV antigen in a single formulation. This approach could permit improved vaccinal efficacy toward a new generation of high-performance vaccines against vector-borne diseases.

The medical importance of *A. aegypti* is clear, such as the failure to control it, the large number of transmitted pathogens, and the persistently high number of cases, as observed in dengue. In this scenario, biotechnological proposals, such as TBV development, could result in an important reduction in number of vectors and prevent the transmission of pathogens.

## Author Contributions

MR-A, OM-J, PS, RM, JL, TS, IS, DL, MM, LR, and RG wrote the manuscript. DS-L, WD, NG, RA, MS, LA, FF, LM, and RG reviewed the manuscript. MR-A, OM-J, NG, RA, MS, LA, FF, LM, and RG drafted and critically evaluated the manuscript. All authors contributed to the article and approved the submitted version.

## Conflict of Interest

The authors declare that the research was conducted in the absence of any commercial or financial relationships that could be construed as a potential conflict of interest.
